# Pyrethroid resistance in the major malaria vector *Anopheles arabiensis *from Gwave, a malaria-endemic area in Zimbabwe

**DOI:** 10.1186/1475-2875-7-247

**Published:** 2008-11-28

**Authors:** Givemore Munhenga, Hieronymo T Masendu, Basil D Brooke, Richard H Hunt, Lizette K Koekemoer

**Affiliations:** 1 Department of Biological Sciences, University of Zimbabwe, P.O. Box MP 167, Mount Pleasant, Harare, Zimbabwe; 2 Vector Control Reference Unit, National Institute for Communicable Diseases, NHLS, Private Bag X4, Sandringham, Johannesburg 2131, South Africa; 3 School of Animal, Plant and Environmental Sciences, University of the Witwatersrand, Johannesburg, South Africa; 4 Department of Public Health, P. Bag F26, Francistown, Botswana; 5 Division of Virology and Communicable Diseases Surveillance, School of Pathology of the National Health Laboratory Service and the University of the Witwatersrand, Johannesburg, South Africa

## Abstract

**Background:**

Insecticide resistance can present a major obstacle to malaria control programmes. Following the recent detection of DDT resistance in *Anopheles arabiensis *in Gokwe, Zimbabwe, the underlying resistance mechanisms in this population were studied.

**Methods:**

Standard WHO bioassays, using 0.75% permethrin, 4% DDT, 5% malathion, 0.1% bendiocarb and 4% dieldrin were performed on wild-collected adult anopheline mosquitoes and F_1 _progeny of *An. arabiensis *reared from wild-caught females. Molecular techniques were used for species identification as well as to identify knockdown resistance (*kdr*) and *ace-1 *mutations in individual mosquitoes. Biochemical assays were used to determine the relative levels of detoxifying enzyme systems including non-specific esterases, monooxygenases and glutathione-S-transferases as well as to detect the presence of an altered acetylcholine esterase (AChE).

**Results:**

*Anopheles arabiensis *was the predominant member of the *Anopheles gambiae *complex. Of the 436 *An. arabiensis *females, 0.5% were positive for *Plasmodium falciparum *infection. WHO diagnostic tests on wild populations showed resistance to the pyrethroid insecticide permethrin at a mean mortality of 47% during February 2006 and a mean mortality of 68.2% in January 2008. DDT resistance (68.4% mean mortality) was present in February 2006; however, two years later the mean mortality was 96%. Insecticide susceptibility tests on F_1 _*An. arabiensis *families reared from material from two separate collections showed an average mean mortality of 87% (n = 758) after exposure to 4% DDT and 65% (n = 587) after exposure to 0.75% permethrin. Eight families were resistant to both DDT and permethrin. Biochemical analysis of F_1 _families reared from collections done in 2006 revealed high activity levels of monooxygenase (48.5% of families tested, n = 33, p < 0.05), glutathione S-transferase (25.8% of families tested, n = 31, p < 0.05) and general esterase activity compared to a reference susceptible *An. arabiensis *colony. Knockdown resistance (*kdr*) and *ace-I*^*R *^mutations were not detected.

**Conclusion:**

This study confirmed the presence of permethrin resistance in *An. arabiensis *populations from Gwave and emphasizes the importance of periodic and ongoing insecticide susceptibility testing of malaria vector populations whose responses to insecticide exposure may undergo rapid change over time.

## Background

Malaria remains the most important parasitic disease in Zimbabwe causing significant mortality and morbidity despite concerted effort to control it. Approximately 50% of the population live in malarious areas and are at risk of infection [1]. The greatest burden of malaria occurs in the low lying areas of the country [2,3], with children under five years of age, pregnant women and people living with HIV and AIDS being the most vulnerable [1].

*Anopheles arabiensis *is the main malaria vector in Zimbabwe [4,5]. *Anopheles funestus *and *Anopheles merus *occur sporadically and may be involved in malaria transmission in isolated incidences. The principal malaria intervention strategies in Zimbabwe include case management, vector control such as indoor residual spraying (IRS), and health education [1]. House spraying remains the Ministry of Health and Child Welfare's (MHCW) principal strategy for malaria vector control and malaria prevention. Indoor residual spraying started in 1949 with the use of benzene hexachloride (BHC) [6]. BHC was replaced with DDT after the discovery of a BHC resistant population of *An. arabiensis *in the lowveld of the country [7]. The malaria control programme was briefly interrupted between 1976 and 1980 due to political unrest. Immediately after independence malaria vector control using DDT resumed. In order to manage bed bug resistance DDT and deltamethrin were used interchangeably for malaria vector and tsetse fly control from 1987 until 1991, when environmentalists succeeded in lobbying against its use. DDT has been re-introduced for adult vector control to complement the pyrethroid arsenal which includes deltamethrin, lambdacyhalothrin and alphacypermethrin.

Insecticide resistance can be defined as a reduction in the insecticide sensitivity of an insect population. This is reflected by repeated failure of an insecticide to achieve the expected level of control when used according to the recommendations for that pest species [8]. Insecticide resistance is generally mediated by behavioural, metabolic or physiological factors and usually results from one or more of three different mechanisms: reduction in insecticide penetration, an increased metabolism of insecticide by metabolic enzymes and or modification of the insecticide target site [9]. Insecticide resistance management becomes complicated if cross resistance or multiple insecticide resistance develops within a species. Multiple insecticide resistance occurs when insects develop resistance to several compounds, limiting the choice of insecticides that can be used.

Resistance management strategies require comprehensive information concerning malaria vector species composition in the area of interest, susceptibility to the insecticides proposed for use for their control and an understanding of the underlying resistance mechanisms [8]. Insecticide susceptibility monitoring has been confined to standard WHO bioassays conducted biannually by the National Institute of Health Research (formerly, Blair Research Laboratory). Despite the long-term use of pesticide in both agriculture and health, there have been few instances when resistance has been recorded. Two cases of resistance have been documented; one in Chiredzi involving BHC [7] and, more recently, DDT resistance in Gokwe [4,10]. An increase in malaria cases, especially in the 2003/4 season, has been attributed to the current socio-economic challenges facing the country resulting in difficulties in IRS coverage [1]. However, despite a marked increase in IRS and ITN coverage between 2004 and 2006, malaria cases, though declining, are still comparatively high. Therefore, factors other than socio-economic challenges might be playing a role. This paper reports on the vector status of *An. arabiensis *and its susceptibility to insecticides in Gokwe.

## Methods

### Mosquito collections

**Figure 1:**
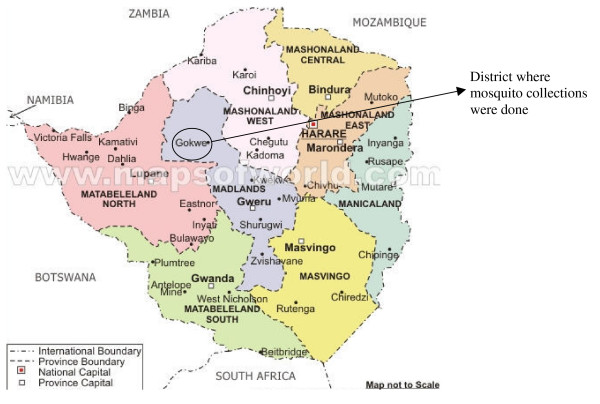
Map of Zimbabwe showing study site location .

Anopheline mosquitoes were collected in Zimbabwe from Gwave (17°55' S; 28°41' E), a village in Gokwe South District, Midland's Province (Figure 1), in February 2006 and January 2008. Adult mosquitoes were captured between 1800 hrs and 2100 hrs using human-baited and cattle-bait net trapping [11] and cattle kraal collections using aspirators. Larvae were collected from stagnant pools at an artesian well and adjacent homesteads and were reared through to adults. Exit window traps were used to collect adults leaving houses [12]. Houses were searched between 0600 hrs and 1000 hrs in order to collect indoor-resting mosquitoes.

### Mosquito processing

Female mosquitoes collected from the field and those reared from larvae were identified morphologically [13], and split into two cohorts. One cohort was used for WHO insecticide susceptibility assays and the other batch was kept alive to obtain F_1 _progeny. Dead specimens were desiccated on blue-indicator silica gel. Specimens were transported to the Vector Control Reference Unit (VCRU), National Institute for Communicable Diseases, Johannesburg, South Africa.

Wild-caught females were blood fed and separated into individual oviposition tubes. Families from each egg batch were reared separately, and 1–3 day old F_1 _female progeny from large isofemale lines were divided into separate samples for bioassay and biochemical analyses. The specimens for biochemical assays were stored at -70°C.

Owing to a lack of statistical correlation between susceptibility to insecticide as determined by bioassay and enzyme levels/activities as determined by biochemical assay in the 2006 collection, a second round of fieldwork was conducted in the same area in 2008. Isofemale lines were set up as described above. However, F_1 _adults showing survival to 4% DDT were pooled and the same was done for the survivors of 0.75% permethrin exposures. These resistant individuals were allowed to mate and their progeny, labelled F_2_, were subsequently used for biochemical analysis.

### Species-specific identification

Wild caught adults were identified to species level. The *An. gambiae *complex sibling species were identified by PCR. [14]. Specimens belonging to the *An. funestus *group were identified using the multiplex PCR assay [15]. Samples which gave hybrid bands using the *An. funestus *multiplex PCR were further assayed with the *Anopheles longipalpis *multiplex PCR [16].

### Sporozoite detection

The head and thoraces of female mosquitoes were tested for the presence of *P. falciparum *using ELISA [17]. A positive control (recombinant *P. falciparum*) and negative control (un-infected female *An. arabiensis *from the Botha DeMellion insectary) were used. Results were scored both visually as well as photometrically at 405 nm using a plate reader (Multiskan RC vl. 5.0, Genesis version 3.03, Labsystems).

### Insecticide susceptibility tests

The standard WHO susceptibility tests were conducted on field collected material using test-kits and insecticide-impregnated filter papers supplied by the WHO. Wild caught adults and 1–3 day old post-emergence adults reared from larval collections were exposed to 4% DDT, 0.75% permethrin, 0.1% bendiocarb, 4% dieldrin, and 5% malathion for one hour. Each test consisted of 25 mosquitoes per tube with two controls. Two to six replicates were performed for each exposure set. For laboratory bioassays, 5 to 25, 3 day old F_1 _progeny were exposed to either 4% DDT or 0.75% permethrin. In all cases insecticide treated papers were tested; both prior to and after the exposures against a susceptible *An. arabiensis *colony (KGB strain) as quality assurance.

For each bioassay, knockdown of mosquitoes was recorded after 60 minutes and final mortality scored after a 24 hour recovery period while supplied with 10% (w/v) sugar solution. Insecticide susceptibility was classified according to the WHO criterion, which considers mortality above 98% and below 80% representative of susceptible and resistant populations, respectively, while the intermediates need further investigation [8].

### Molecular assay for knockdown resistance

Mutations associated with knockdown resistance to pyrethroids and/or DDT were assayed in randomly selected bioassay survivors of wild caught females as well as those females where F_1 _progeny showed resistance to both DDT and pyrethroids using the standard PCR assay [18,19] Due to lack of repeatability the assay was adapted as follows: Three independent PCR assays were set up for each sample: the first contained the primers Agd 2 (5' AGACAAGGATGATGAACC 3') and Agd 4 (5' CTGTAGTGATAGGAAATTTA 3') which amplify a 137-bp product for the insecticide susceptible allele; the second contained primers Agd 1 (5'ATAGATTCCCCGACCATG 3') and Agd 3 (5' AATTTGCATTACTTACGACA 3') for amplifying a 195-bp product associated with the West African resistant allele and the third contained primers Agd1 and Agd5 (5' TTTGCATTACTTACGACTG 3') which amplify a 195-bp product associated with the East African resistant allele [19]. PCR conditions were as previously described [20]. Reference standards were as follows: the positive control consisted of a DNA template from mosquitoes with known West African allele (*kdr-w*) i.e SENN-DDT (homozygous resistant, RR), while the negative control had KGB (homozygous susceptible, SS). No colony with the East African allele (*kdr-e*) was available. A DNA-free reaction mixture was used as a blank in all cases.

### Sequence analysis of the IIS6 domain

The 293-bp fragment of the IIS6 domain spanning the *kdr *mutation site was amplified using primers Agd1 and Agd2 in 38 mosquitoes inclusive of six specimens which showed cross resistance to both DDT and permethrin exposure. Amplicons were sequenced by Inqaba Biotechnical industries, South Africa. The DNA sequences were analysed with Lasergene software (DNASTAR version 7; SeqMan programme, Inc, Madison, WI). Basic Local Alignment Search Tool (BLAST) was used to confirm that the correct gene fragment had been amplified and sequenced [21].

### Ace-1^R ^mutation

A PCR diagnostic test was used to detect the presence of the G119S mutation in seven specimens which showed evidence of altered AChE during biochemical assays using the method described by Weill [22] with modifications. Briefly, the ace-1^R ^gene was amplified by PCR with F (5' CCGGGCGCGACCATGGAA 3') and R (5' ACGATCACGTTCTCCTCCGA 3') oligonucleotide primers. The reaction was performed in 12.5 μl volume containing 1.25 μl of 10× buffer, 200 μM dNTP, 0.1U *Taq *polymerase, 10 pmol of each primer and 1–10 ng of extracted DNA. PCR cycling conditions included an initial denaturation step at 94°C for 2 mins, followed by 40 cycles of: denaturation at 94°C for 30 secs, annealing at 53°C for 30 secs and primer extension at 72°C for 1 min. These cycles were followed by a final auto extension at 72°C for 5 mins. Five microlitres of the amplicons were electrophoresed on a 2.5% agarose gel and visualized under an ultraviolet transilluminator to confirm whether the expected band size had been amplified while the remainder of each amplicon was sent to Inqaba Biotechnical Industries, South Africa for sequencing. Sequences were analysed with Lasergene software as previously outlined. Controls consisted of *An. arabiensis *individuals from families which did not show AChE activity during biochemical assays.

### Biochemical analysis

Levels of monooxygenase, non-specific esterase, glutathione-S-transferase (GST) and altered acetylcholine esterase (AChE) were assayed from individual 1–3 day old post emergence mosquitoes, using cohorts of 47 mosquitoes per microtitre plate [23]. From each family, 6–10 female mosquitoes were assayed with several families/plate in comparison to 11 specimens of a susceptible *An. arabiensis *(KGB) strain. For F_2 _progeny, 24 females were assayed on the same plate as 24 susceptible colony *An. arabiensis*. Mean enzyme activities measured as optical densities at specified wavelengths were compared between the families and samples from the susceptible reference colony using two sample t-tests of means following adjustment for total protein content (STATISTIX 7.0; Tallahassee, FL, USA). Significance in difference was determined at p < 0.05.

## Results

### Mosquito collections

**Figure 2:**
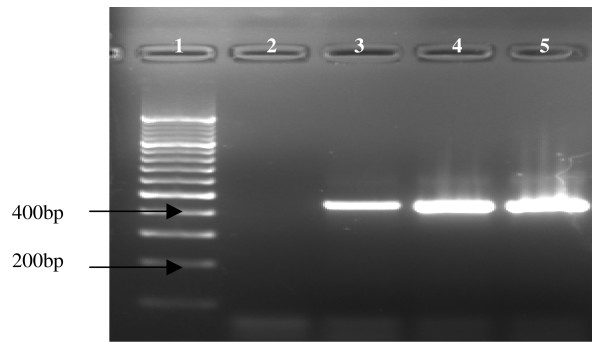
Multiplex PCR to identify *An. longipalpis *[16]. Lane 1: 1 Kb molecular weight marker, Lane 2: Negative control, Lane 3: *An. longipalpis *Type C (positive control), Lane 4 and 5: *An. longipalpis *Type C.

In total, 924 anophelines belonging to four different taxa were collected and positively identified (Table 1). Three members of the *An. gambiae *complex occurred in sympatry. *Anopheles arabiensis *was the predominant species contributing 73.4% to the total collection, while *An. merus *(14%) and *Anopheles quadriannulatus *(11.4%) were the other species collected. Other taxa collected included members of the *Anopheles squamosus *group (0.6%), *Anopheles coustani *(0.3%) and *An. funestus *group (0.2%). Two specimens morphologically identified as *An. funestus *were subsequently shown to be *An. longipalpis *following the multiplex PCR assay [16,24] (Figure 2).

**Table 1:** Summary of anophelines caught and positively identified in Gwave (MBN = "man-baited net trap" and N = Sample size)

Collection period	Collection Method	N	*An. arabiensis*	*An, quadriannulatus*	*An. merus*	*An. longipalpis*	*An. squamosus*	*An. coustani*
February, 2006	Cattle kraal	511	422	66	12	2	6	3
	Larval	43	36	6	1	0	0	0
	MBN	22	12	1	9	0	0	0
January, 2008	Cattle bait	181	101	7	73	0	0	0
	Larval	144	85	25	34	0	0	0
	Window exit	23	23	0	0	0	0	0
Totals (%)		924	679(73.4)	105(11.4)	129(14.0)	2(0.2)	6(0.6)	3(0.3)

### Mosquito infectivity

A total of 530 anophelines were assayed for infection with *P. falciparum *sporozoites (436 *An. arabiensis*, 73 *An. quadriannulatus *and 21 *An. merus*). All were negative except for two infected *An. arabiensis *giving an overall infection rate of 0.5% for this species. Both specimens were collected using man baited net traps (MBN) during 2006.

### Insecticide susceptibility tests

Table 2 shows the results of standard WHO susceptibility tests against wild caught mosquitoes. *Anopheles arabiensis *is resistant to permethrin, but completely susceptible to malathion, dieldrin and bendiocarb. Samples collected in February 2006 showed high resistance to DDT (68.4%) while those collected in January 2008 showed the population to be almost susceptible (96%). Exposure to 0.75% permethrin of families reared from *An. arabiensis *collected during 2006 showed evidence of resistance in 21 families (56.8%, n = 37) with mortalities ranging from 0% to 100% with an average of'69.8% across the families. Permethrin exposures of F_1 _families reared from samples collected in January 2008 showed resistance in 11 families (78.6%, n = 14). Final mortalities following exposure to 4% DDT ranged from 28.6% to 100%. There was evidence of resistance in 16 families (25.4%, n = 59) in samples collected in 2006 and 2 families (14.3%, n = 14) in samples collected in 2008. Table 3 summarizes results of families exposed to both insecticides. Eight families (six collected in 2006 and two collected in 2008) showed resistance to both DDT and permethrin.

**Table 2:** Field susceptibility tests carried out on *An*. *arabiensis *caught in Gwave in February, 2006 and January, 2008 (* Resistant according to WHO criteria).

	Insecticides									
	0.75% Permethrin (Pyrethroid)		4% DDT (Organochlorine)		4% dieldrin (Cyclodienes)		0.1% bendiocarb (Carbamate)		5% malathion (Organophosphates)	
Collection period	Total (n)	(%) mort	Total (n)	(%) mort	Total (n)	(%) mort	Total (n)	(%) mort	Total (n)	(%) mort
February, 2006	87	47*	110	68.4*	37	100	40	100	52	100
January, 2008	66	68.18*	75	96*	-	-	100	98.9	-	-

**Table 3:** WHO insecticide susceptibility test results on 1–3 day old F_1 _*An. arabiensis *reared from females collected from Gwave village in 2006 and 2008. Results expressed as % mortality 24 hr post exposure. (* indicate Families showing cross resistance to DDT and permethrin, Fam = Family)

**February 2006**														
	4% DDT		0.75% permethrin			4% DDT		0.75% permethrin			4% DDT		0.75% permethrin	
Fam.	(n)	% mort	(n)	% mort	Fam.	(n)	% mort	(n)	% mort	Fam.	(n)	% mort	(n)	% mort
27*	7	28.6	13	53.9	82	9	100	8	63.6	113	10	60	4	100
37	11	72.7	11	81.8	84	10	90	11	54.5	118*	9	66.7	6	16.7
38	9	100	12	66.7	85	13	100	10	50	120	10	100	10	100
46	5	100	8	0	90	8	100	10	100	135	11	100	10	80
48	10	100	10	70	94*	8	62.5	9	44.4	139	11	100	6	66.7
53	11	100	12	100	95	13	69.2	6	100	144*	14	78.6	15	66.7
57	7	100	15	46.7	97	11	81.8	13	92.3	149	11	54.6	10	100
64*	12	33.3	8	50	101	9	100	11	18.2	156*	13	69.2	11	45.5
66	7	100	11	45.5	103	9	100	10	90	169	10	100	10	60
74	12	16.7	12	100	104	8	87.5	12	50	166	8	100	12	83.3
77	14	100	11	63.6	108	7	100	8	100	178	7	100	9	55.6
80	10	100	10	90	111	11	100	11	72.7	180	9	100	9	100

**January 2008**														
	4% DDT		0.75% permethrin			4% DDT		0.75% permethrin			4% DDT		0.75% permethrin	
Fam.	(n)	% mort	(n)	% mort	Fam.	(n)	% mort	(n)	% mort	Fam.	(n)	% mort	(n)	% mort

654	11	100	15	93.33	740	16	100	18	72.2	821	12	100	15	53.3
681	14	100	13	61.54	755	13	100	20	55	854	15	100	15	100
682*	25	74	19	68.23	769	23	100	21	52.4	856	10	100	8	50
712	12	100	15	40	771*	10	78	14	14.3	858	15	100	15	100
738	11	100	10	20	808	20	100	17	35.3					

### Detection of *kdr *alleles

Molecular analysis of 54 individuals by allele-specific PCR showed the presence of both the East and West African *kdr *mutations. All three genotypes determined by *kdr *PCR (RR, RS and SS) were detected in insecticide bioassay survivors as well as susceptibles. However, sequence analysis of the region spanning the *kdr *mutation in 38 of these individuals showed a complete absence of both the "Leu-Phe" and "Leu-Ser" *kdr *mutations.

### Ace-1^R ^mutation

The *ace-I*^*R *^mutation was not detected in any of the seven specimens sequenced. Therefore, the reduced sensitivity of AChE activity to propoxur inhibition as detected biochemically in some families cannot be attributed to the single point mutation G119S (gene ace-*I*) [22].

### Biochemical assays

**Figure 3:**
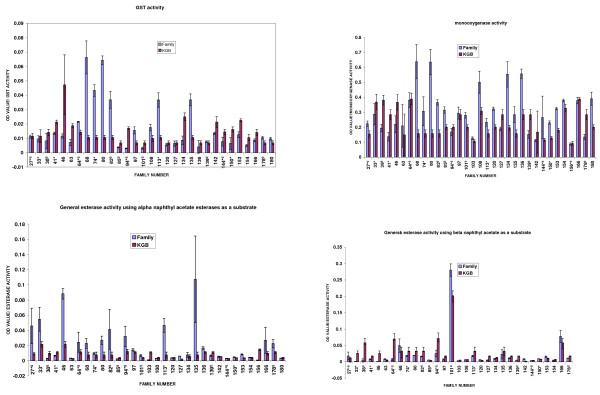
Mean optical density values of GST, monooxygenase and esterases enzymes in *An. arabiensis *F_1 _progeny reared from collections done in 2006, by family, and corresponding activity for susceptible *An. arabiensis *(KGB) samples assayed simultaneously.

Adults reared from F_1 _progeny were biochemically analysed for comparative enzyme levels. Figure 3 shows the average levels of GST, monooxygenase and esterase activities for F_1 _progeny in *An. arabiensis *families. Eight families (25.8%, n = 31) showed significantly higher levels of GST activity (P < 0.05) compared to the reference KGB sample. Average levels of non-specific esterase activity/family (using α-naphthyl acetate as substrate) indicated nine families (27.3%, n = 33) with significantly higher levels of esterase activity (p < 0.05) than their corresponding KGB control samples. There was a significant correlation between esterase level and permethrin as well as DDT bioassay mortality data (p < 0.05). Assays using β-naphthyl acetate as a substrate showed that only two families (6.5%, n = 31) had significantly increased esterase activity. Levels of monooxygenase were significantly elevated in 16 families (48.5%, n = 33). There was no significant correlation between monooxygenase activity and bioassay (permethrin and DDT) mortality data (p > 0.05). Figure 5 shows the mean percentage propoxur inhibition of AChE using F_1 _progeny reared from the 2006 collections. Only seven families (20.6%, n = 34) showed evidence of an altered AChE based on the criterion that enzyme inhibition of less than 70% indicates significantly reduced AChE sensitivity to propoxur [23].

**Figure 4:**
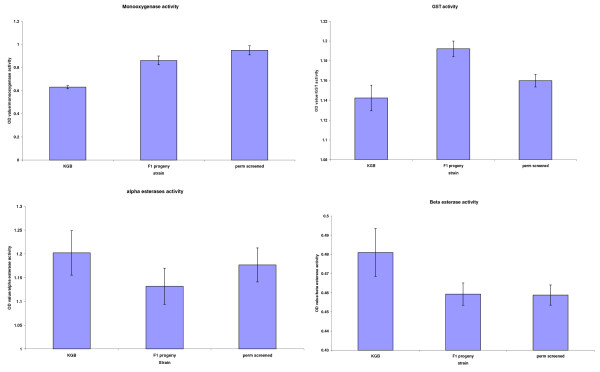
Mean optical density values of GST, monooxygenase and esterases enzymes of F_1 _and F_2 _*An. arabiensis *progeny (perm screened) reared from collections done in 2008 and corresponding activity for susceptible *An. arabiensis *(KGB) samples assayed simultaneously.

The lack of statistical correlation between the bioassay and biochemical assays may have been due to the presence of susceptible individuals in each family resulting in the masking of the elevated enzyme levels. It was therefore decided to characterize the resistance mechanism further by allowing F_1 _bioassay survivors from the 2008 collections to mate and produce F_2 _progeny, thereby preventing the need to colonise *An. arabiensis *and then artificially select for either pyrethroid or DDT resistance. The F_2 _generation would then theoretically be composed of resistant individuals and would reduce the masking effect of susceptible siblings. Biochemical analysis on F_2 _adults showed a significant elevation in monooxygenase, (p < 0.05) activity when compared to the susceptible reference colony (KGB) (Figure 4). There was also a marked increase in monooxygenase activity in the F_2 _cohorts compared to the F_1 _progeny.

**Figure 5:**
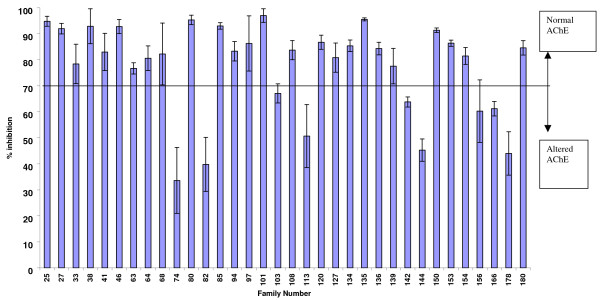
Mean acetylcholinesterase percentage inhibition by propoxur in F_1 _progeny of *An. arabiensis *reared from wild-caught females collected in Gwave during February 2006.

## Discussion

Malaria vector composition and distribution in Zimbabwe is well documented. In Gokwe, members of the *An. gambiae *complex, predominantly *An. arabiensis*, have been implicated as the main vectors [4,5]. This study confirms *An. arabiensis *as the main malaria vector in Gwave. *Anopheles arabiensis *predominated over all the other sibling species in wild collections and was the only species found to be infected with the malaria parasite *P. falciparum*. The low infectivity rate detailed here could be attributed to the timing of mosquito collections as malaria transmission peaks from mid February to late April in Zimbabwe [5]. The presence of *An. longipalpis *Type C in our collections, which we had morphologically mis-identified as *An. funestus*, highlights the need for molecular species identification, especially where malaria vectors occur in sympatry with closely related non-vectors.

The insecticide susceptibility status of malaria vectors in Zimbabwe remains unclear. Some reports detail complete susceptibility to insecticides in Gokwe [25,26] whilst another reported DDT resistance from the same area [4]. In order to understand the resistant mechanisms that might be involved, insecticide susceptibility, molecular and biochemical assays were used. The importance of continual insecticide resistance monitoring is clearly illustrated by the difference in susceptibility to DDT between the two collection periods. Patterns of insecticide resistance may vary considerably in time and it is therefore important to have an active entomological surveillance system as part of a malaria vector control programme. From the surveys detailed here, resistance to permethrin was consistently recorded while the population showed complete susceptibility to organophosphates and carbamates.

Elevated GST activity is often associated with DDT resistance in insects where resistance is achieved by dehydrochlorination of DDT to DDE [27]. In data presented here, GST activity was significantly elevated in eight families reared from wild caught females, providing a strong candidate mechanism for production of the resistance phenotype. *Anopheles arabiensis *resistance to DDT in this locality was first reported during a survey carried out between 1999 and 2002 [4], and was attributed to high usage of organochlorines by villagers as well as a long history of DDT usage in this area. DDT was used as far back as 1970 when it was used for both tsetse fly and malaria vector control [28]. Currently DDT is being used interchangeably with pyrethroid insecticide (Icon^®^). The Icon^® ^might have reduced the selective pressure imposed by DDT resulting in an increase in DDT susceptible individuals in the specimens collected during 2008. This probably explains the variation in DDT susceptibility.

Pyrethroid resistance is often mediated by monooxygenase detoxification. Resistance to permethrin in the samples tested here may be attributed to increased levels of monooxygenase titres as recorded in the F_1 _progeny of 16 wild caught *An. arabiensis *females. Selection for permethrin resistance in the F_1 _progeny was followed by an increase in monooxygenase as well as general esterase activity. The combined effect of elevated monooxygenase and esterase activity in permethrin resistant *Anopheles *mosquitoes has previously been reported [29,30].

This is the first instance of pyrethroid resistance recorded in a malaria vector species in the Gokwe district. Such resistance has serious implications for malaria control, considering that deltamethrin and lamdacyhalothrin are the main insecticides currently being used for vector control by the Zimbabwe National Malaria Control Programme. These insecticides are also recommended by WHO for vector control, especially for bed net treatment. The development of resistance may be due to selection pressure from agricultural use of pyrethroids. For example, the local community uses pyrethroids for controlling cotton pests. A survey from 1999 showed the high rank of pyrethroids among the insecticides used in Gokwe [4,10]. Residues of pyrethroids sprayed on cotton and rice crops have been suggested as the source of selection favouring the emergence of pyrethroid resistance [31,32].

The occurrence of resistance of both DDT and permethrin observed during 2006 is a strong indicator of the presence of target site insensitivity. Target site insensitivity, sometimes known as knock-down resistance (*kdr*), has been closely associated with cross-resistance to DDT and pyrethroids in malaria vectors [19,20,33,34]. Results presented here show a complete absence of both East and West African *kdr *mutations in those families showing resistance to both DDT and pyrethroids. It is conceivable that alternative amino acid substitutions in the *An. arabiensis *sodium channel gene may be responsible for resistance to DDT and pyrethroids. Based on the discrepancy between PCR and sequence data this study confirms the widely held view that mutation-specific PCR assays developed to detect single nucleotide polymorphisms are often difficult to optimize and may not be as reliable as other methods [35,36]. The inconsistent PCR results obtained after repeating the assay using the same samples under similar conditions as well as the disparity between sequence data and PCR results has previously been reported from this laboratory. During an investigation of pyrosequencing as an alternative for detecting *kdr *mutations it was shown that PCR results did not correlate with either sequence or pyrosequence data (Vezenegho, unpublished data). A poor correlation between PCR and sequence data has also been demonstrated for *An. arabiensis *in Sudan [37].

## Conclusion

This study confirmed the presence of DDT and permethrin resistance in *An. arabiensis *in Gokwe. Both resistance phenotypes are most likely based on metabolic detoxification. The way forward is careful consideration on the use of insecticides. A mosaic system of insecticide application or rotational use of insecticides to slow the spread of DDT and pyrethroid resistance is suggested. We also recommend regular monitoring of resistance using WHO bioassays.

## Competing interests

The authors declare that they have no competing interests.

## Authors' contributions

GM carried out field work, species-specific identification, ELISAs, biochemical assays, interpretation of all results and wrote the first and subsequent drafts of the manuscript.

HTM was involved in project design and field work. BDB helped interpret the results of the biochemical assays and contributed to the writing of the manuscript. RHH was involved in field work, morphological identification of anophelines and provided comments on the manuscript. LLK conceived the project, oversaw its implementation, carried out laboratory bioassays, assisted with species identification and contributed to the subsequent writing of the manuscript.
